# CT perfusion abnormalities in status epilepticus: associations with clinical characteristics, EEG, and outcome measures

**DOI:** 10.1007/s00415-026-13886-y

**Published:** 2026-06-15

**Authors:** Leyla Baysal, Jonas Böhm, Monika Huhndorf, Ulf Jensen-Kondering, Naomi Larsen, Nils G. Margraf

**Affiliations:** 1 Epilepsy Center, Department of Neurology, University Hospital Schleswig-Holstein, Campus Kiel, Christian-Albrechts-University, Arnold-Heller-Str. 3, 24105 Kiel, Germany; 2 Department of Neuroradiology, University Hospital Schleswig-Holstein, Campus Kiel, Arnold-Heller-Str. 3, 24105 Kiel, Germany; 3 Department of Neuroradiology, University Hospital Schleswig-Holstein, Campus Lübeck, Ratzeburger Allee 160, 23538 Lübeck, Germany

**Keywords:** Seizure-related perfusion changes, Perfusion imaging, Stroke mimics, Lateralized periodic discharges, Emergency neuroimaging

## Abstract

**Objective:**

To investigate cerebral perfusion patterns on computed tomography perfusion (CTP) imaging in patients with status epilepticus (SE) and to evaluate their association with clinical, electrophysiological, and outcome parameters.

**Methods:**

We conducted a retrospective study of 117 patients (mean age 74.5 ± 12.1 years, 63 females) who underwent emergency CTP due to focal neurological deficits and suspected acute stroke; all patients were ultimately diagnosed with SE. Periictal CTP findings were analyzed and correlated with clinical characteristics, laboratory results, electroencephalographic (EEG) patterns, and patient outcomes.

**Results:**

Normoperfusion was the most common CTP finding (*n* = 54, 46.2%), followed by hypoperfusion (*n* = 36, 30.8%) and hyperperfusion (*n* = 27, 23.1%). Patients with CTP hyperperfusion had significantly longer hospital stays and longer SE duration (*p* < 0.05). Hyperperfusion was also more frequently associated with ictal EEG activity or lateralized periodic discharges (LPDs). Perfusion abnormalities outlining the boundaries of vascular territories were striking findings, observed in 80.9% of our patients with SE. At discharge, 70 patients (59.8%) had an unfavorable outcome, including 26 deaths (22.2%). Longer SE duration and poor treatment response were independent predictors of mortality. CTP abnormalities were not associated with SE etiology, seizure semiology, or overall functional outcome.

**Conclusion:**

CTP may serve as a useful tool for early triage in centers with limited neurophysiological resources and may help suggest nonconvulsive SE in diagnostically unclear cases. Hyperperfusion on CTP appears to reflect disease severity and prolonged seizure activity rather than brain injury. Ictal EEG patterns can be associated with both hyperperfusion and hypoperfusion. CTP abnormalities are often associated with boundaries that are not confined to vascular territories. These findings underscore the importance of integrating imaging and electrophysiological data to improve the early diagnosis and management of SE.

**Supplementary Information:**

The online version contains supplementary material available at 10.1007/s00415-026-13886-y.

## Introduction

Status epilepticus (SE) is a neurological emergency associated with substantial morbidity and mortality, and early treatment is critical to reduce the risk of poor outcomes [1]. Diagnostic evaluation is particularly challenging in patients who present with prolonged alterations of consciousness without overt motor manifestations, behavioral changes (including agitation, delirium, or speech disturbances), and subtle motor phenomena, i.e., nonconvulsive status epilepticus (NCSE). In the emergency department, these clinical features may closely mimic acute ischemic stroke, leading to potential misdiagnosis [2]. Rapid detection of SE is crucial to enable the immediate initiation of antiseizure medication (ASM). Although electroencephalography (EEG) is regarded as the diagnostic gold standard for NCSE [3], its immediate availability in emergency settings is often limited. EEG interpretation in this context can be challenging, as ambiguous patterns such as those within the ictal–interictal continuum may be observed [4].

Periictal neuroimaging abnormalities are transient imaging changes associated with seizure activity and SE. Periictal magnetic resonance imaging (MRI) abnormalities (PMA)—including diffusion restriction and FLAIR hyperintensities and increased perfusion on arterial spin labeling (ASL)—have been reported in a substantial proportion of SE patients [5–7]. These findings may serve as an early biomarker of seizure-related structural alterations and indirectly reflect metabolic changes during sustained cortical activity [5]. Previous studies have shown that PMA most commonly involves the neocortex, particularly the frontal lobes, and is typically unilateral. These imaging abnormalities correlate well with focal EEG findings, especially lateralized periodic discharges (LPDs) [6, 8]. Importantly, the presence of PMA has been associated with poor clinical outcome, including refractory SE, in-hospital mortality, and delayed-onset epilepsy [5].

While MRI-based findings are increasingly recognized, less is known about perfusion imaging, particularly regarding computed tomography perfusion (CTP). CTP is frequently used in the acute setting for suspected stroke and represents a rapid and widely accessible alternative, especially in emergency situations [7].

Changes in cerebral perfusion are a well-established feature of epileptic activity and have primarily been documented in human studies using single photon emission computed tomography (SPECT) and ASL-MRI [9, 10]. CTP is a well-established and rapidly accessible tool in acute stroke diagnostics and may, therefore, represent a valuable adjunct in the evaluation of suspected SE. Since cerebral perfusion is closely linked to neuronal activity, areas of hyperperfusion on CTP imaging may serve as an indirect marker of ongoing epileptic activity [11]. Previous studies have reported heterogeneous perfusion patterns in patients with convulsive or NCSE, including normoperfusion, hypoperfusion, and hyperperfusion [12, 13]. However, the diagnostic utility of CTP remains constrained by the absence of standardized perfusion thresholds, the lack of knowledge on the time course of perfusion impairment, and considerable interrater variability. Furthermore, the prognostic relevance of periictal perfusion abnormalities has not yet been sufficiently elucidated.

The aim of the present study is to evaluate the diagnostic significance of periictal CTP abnormalities in patients with SE and to investigate their association with clinical features, laboratory findings, electrophysiological patterns, and patient outcomes. We hypothesize that CTP may aid in the identification of stroke mimics such as SE and could be integrated into diagnostic algorithms for patients presenting with suspected epileptic emergencies.

## Methods

We retrospectively analyzed patients diagnosed with SE at the University Hospital Schleswig–Holstein (UKSH), Campus Kiel, from January 2019 to December 2024. Patients were eligible for inclusion if their SE diagnosis was clinically confirmed by a neurologist experienced in epileptology following an initial emergency department evaluation.

### Study design

We identified all consecutive adult patients with a primary diagnosis of SE who received CTP imaging during their hospitalization via the institutional database. Eligible cases were limited to individuals aged over 18 years, identified using the International Classification of Diseases, 10th Revision (ICD-10) code G41. The ethics committee of the medical faculty of the Christian-Albrechts-University, Kiel approved the retrospective scientific use of the clinically acquired data (D465/25). The study was therefore performed in accordance with the ethical standards laid down in the 1964 Declaration of Helsinki and its later amendments.

Our institution maintains 24/7 neurological and neuroradiological coverage. Since CTP is an integral component of the acute stroke diagnostic protocol at our center, it remains available at all times. Patients underwent an immediate CTP examination following the clinically based examination, which was followed by EEG recordings, mostly performed within 2 days. All patients were recruited from our interdisciplinary emergency department. CTP was not performed to establish the diagnosis of SE but was obtained in the acute setting when the initial clinical presentation was unclear. In most cases, patients presented to the emergency department with focal neurological deficits or other symptoms raising concern for acute ischemic stroke. Only patients with a final diagnosis of SE, established based on clinical assessment and/or EEG findings, were included in the analysis.

Only cases that met the 2015 International League Against Epilepsy (ILAE) Task Force criteria for the classification of SE were included. SE was defined as continuous clinical seizure activity lasting longer than 5 min for a tonic–clonic seizure, or more than 10 min for a focal seizure with impaired consciousness or an absence seizure [14]. The ILAE operational classification of SE was applied to categorize the semiology of SE based on the available clinical and EEG data [14]. Seizure events were retrospectively classified through a meticulous review of electronic medical records. This was supplemented by an analysis of documented medical histories obtained directly from patients or from third-party witnesses, as well as paramedical and medical reports describing the suspected ictal events. If the clinical presentation included tonic–clonic or focal motor semiology, the episode was classified as SE with prominent motor symptoms in accordance with the 2015 ILAE classification [14]. Patients with anoxic brain damage due to cardiorespiratory arrest were excluded. Other exclusion criteria were psychogenic nonepileptic seizures or other neurological conditions that would preclude a diagnosis of SE such as toxic or metabolic encephalopathy, movement disorders, syncope with convulsive movements, and patients lacking sufficient neuroimaging data.

Clinical data were extracted from patients’ medical records, including demographics, clinical history, neurological examination findings, Glasgow Coma Scale (GCS) at admission, level of consciousness at onset (dichotomized in the analysis into alert-somnolent or stuporous–comatose), seizure details, SE semiology, family history of epilepsy, baseline functional status [including the modified Rankin Scale (mRS) score], blood tests, cerebrospinal fluid (CSF) analysis, neuroimaging findings, EEG parameters, comorbidities, potential SE causes, acute hospital complications [15], therapeutic management, therapy response, and length of hospital stay. Initial lactate concentrations (reference 0.5–2.2 mmol/L at 37 °C) were measured via venous blood gas analysis within 30 min of hospital admission. Only the baseline measurement from the point-of-care gas analysis was utilized for subsequent statistical evaluation.

To estimate mortality risk, components of the Status Epilepticus Severity Score (STESS) prognostic score were included in the analysis (STESS-3: cut-off level to predict poor outcome ≥ 3 points) [16]. The duration of SE, defined as time period between SE onset and its resolution, was determined based on clinical observation or, in patients with NCSE, according to electrographic seizure activity on EEG. Resolution was defined as normalization of the clinical condition and/or cessation of seizure activity on EEG [17]. Patients were categorized into three groups according to SE duration: less than 24 h, 24–72 h, and more than 72 h. Finally, we conducted an analysis to identify which clinical characteristics were independently associated with in-hospital mortality and functional status at discharge, as assessed by the Glasgow Outcome Scale (GOS) [18]. We also determined the time interval from hospital admission to the onset of the CTP and EEG study.

Etiologic categories were classified in accordance with ILAE recommendations as acute, remote, progressive, or unknown, and were further grouped into broader categories [14, 19, 20]. Four subcategories of acute etiologies were defined according to Lattanzi et al. (2023): acute-triggering factors (withdrawal, low levels, or inappropriate prescription of ASMs, febrile illnesses, or sleep deprivation in patients with preexisting epilepsy), acute primary central nervous system (CNS) pathology (cerebrovascular disease (CVD), active CNS infection, or head trauma), secondary CNS pathology (metabolic disturbances, systemic infection or fever), and acute toxic (drug or alcohol intoxication and withdrawal) [21]. Etiologies that could potentially lead to death were considered fatal, such as severe systemic infections, malignant brain tumors, and acute large-volume intracerebral hemorrhage [22]. Patients who had cerebral lesions likely to be causally related to epilepsy including severe leukoaraiosis were classified as structural epileptogenic lesions [23–25]. Most patients were treated using a stepwise protocol in accordance with the current clinical guidelines [15, 26]. ASMs for SE include a first line with intravenous (IV) benzodiazepines, followed by a second line of IV levetiracetam, valproate, lacosamide, or phenytoin whenever the seizures persisted. Finally, IV anesthetics were used to achieve seizure control in therapy-refractory patients [15, 26]. Refractory status epilepticus (RSE) was defined as SE that did not respond to adequate administration of first- and second-line ASMs [27]. Super-refractory SE (SRSE) was defined as SE that persisted or recurred 24 h or more after the initiation of anesthetic therapy [27].

### EEG

All EEGs were digitally recorded using the international 10–20-electrode placement system with a 21-electrode array, with a duration of 20–30 min. Although continuous EEG monitoring was not accessible, routine EEG studies were repeated on separate days for patients who showed no response to ASMs. EEGs were reviewed and reevaluated by board-certified, experienced epileptologists (NM, LB). The EEG findings for each patient were classified according to the American Clinical Neurophysiology Society (ACNS) standardized critical EEG terminology [28].

The diagnosis of NCSE was done on clinical and EEG grounds by the neurologist and the epileptologist. The Salzburg consensus criteria (SCC) were used for the diagnosis of SE. Subsequently, all included cases of NCSE underwent retrospective reevaluation of their EEGs, with patterns reclassified according to the SCC for NCSE [3]. EEG patterns were classified based on the non-epileptiform abnormalities, interictal epileptiform discharges, and ictal EEG patterns consistent with SE.

### Imaging

All patients underwent nonenhanced cranial CT (NCT), CT angiography (CTA), and CTP of the brain for suspected acute stroke within 2 h of emergency admission on a 128-slice dual-layer SCT system (IQon Spectral CT or Spectral CT 7500, Philips Healthcare, Best, The Netherlands). MRI, including diffusion-weighted, T2-weighted, and fluid-attenuated inversion recovery (FLAIR) sequences, was performed after admission in MRI-compatible patients when clinically indicated to evaluate structural epileptogenic lesions. None of the patients underwent ASL.

All CTP studies were independently reevaluated by two board-certified neuroradiologists (MH, UJK), each with more than 10 years of experience. They were blinded to the patients’ clinical and EEG information. After a consensus reading, disagreements were resolved by a third rater (board-certified neuroradiologist, non-author). We performed a qualitative visual analysis to identify perfusion differences across hemodynamic maps, as this approach is practical for routine clinical use and because of the lack of quantitative thresholds on standard perfusion maps [11, 29, 30].

CTP imaging was performed using a standardized stroke protocol. Acquisition parameters included a tube voltage of 120 kV with an automated mAs product of 21, collimation of 0.625 mm, and a slice thickness of 10 mm. IV contrast administration consisted of 40 mL of iodinated contrast medium (300 mg I/mL; Bracco Imaging S.p.A., Milan, Italy), followed by a 30 mL saline flush, both injected at a flow rate of 4.5 mL/s. CTP scan started with a delay of 5 s after the beginning of contrast injection. Perfusion data were automatically computed without manual input using RAPID software (iSchemaView, Menlo Park, CA, USA) to generate color-coded perfusion maps, including time-to-maximum (Tmax), relative cerebral blood flow (rCBF), relative cerebral blood volume (rCBV), time to peak (TTP), and mean transit time (MTT). CTP studies were evaluated exclusively by visual analysis. Perfusion patterns were classified as hyperperfusion, hypoperfusion, or normoperfusion [31]. A relative increase in rCBF or rCBV, or a decrease in Tmax or TTP (or a combination thereof), in tissue within one hemisphere compared to the contralateral hemisphere was defined as a hyperperfusion pattern. Conversely, a deviation of these parameters in the opposite direction was defined as a hypoperfusion pattern. A vascular origin of the perfusion abnormalities (occlusion, stenosis, vessel variant) was ruled out on CTA imaging. Additionally, restriction to a physiological vascular territory—or the lack thereof—was assessed, along with cortical and thalamic perfusion characteristics. Hyperperfusion detected on CTP was not used as a criterion for diagnosing SE.

### Statistics

Intergroup differences were analyzed using Fisher’s exact or Chi-square for categorical data, and Student’s *t* test or Mann–Whitney *U* tests for continuous data, following normality testing. A *p* value < 0.05 was considered statistically significant. Predictors of poor outcome and treatment refractoriness were evaluated via binary logistic regression, incorporating all significant univariate variables. Interobserver reliability between the two reviewers was calculated, using Cohen’s Kappa (range 0–1.0). All analyses were conducted using Statistical Package for Social Sciences (SPSS) software (v18.0). Bonferroni correction was used for multiple comparisons.

## Results

### Status epilepticus demographics and clinical characteristics

**Fig. 1:**
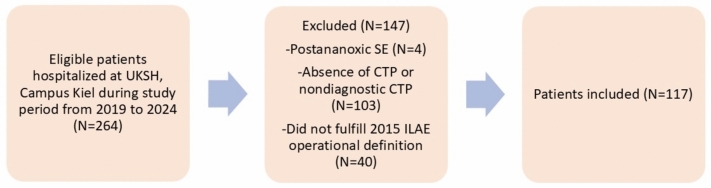
Study flowchart: Among the excluded patients, 103 did not undergo computed tomography perfusion (CTP) or had nondiagnostic CTP due to artifacts. Four patients with a history of resuscitation and cerebral hypoxia were excluded. Additional 40 patients were excluded due to an uncertain diagnosis of status epilepticus (SE), including cases of psychogenic nonepileptic seizures, encephalopathy, or recurrent seizures not meeting the International League Against Epilepsy (ILAE) operational definition of SE [14]

During the study period, 264 patients aged 18 years or older with an SE (ICD Codes G41) were admitted to the neurology department. After excluding 147 admissions that did not meet eligibility criteria, 117 consecutive patients (mean age 74.5 ± 12.1, 63 females) diagnosed with SE and meeting inclusion criteria were enrolled (Fig. 1).

The indication for obtaining a CTP was most often the presence of focal neurological symptoms most frequently involving limb strength and speech (*n* = 83, 70.9%). None of the patients presented with acute stroke. The clinical characteristics of the patients, as well as imaging, laboratory, and EEG findings, are summarized in Table 1. More than half of the patients (*n* = 88, 75.2%) presented with SE with prominent motor symptoms, while 29 (24.8%) patients were diagnosed with only NCSE (Table 1). Motor SE was developed into NCSE in 45 (38.5%) patients. SE etiology was acutely symptomatic in 48 (43.2%) and remote unprovoked in 52 (44.4%) of the patients. The most common presumed etiologic factors were CVD, identified in 53 patients (45.3%), cerebral tumors in 17 (14.6%) patients and head trauma in 12 (10.3%) patients (Table 1). Among SE episodes with acute symptomatic etiology, the subcategories were acute secondary CNS pathology in 27 (56.3%) patients, acute toxic etiologies in 13 (27.1%) patients, acute triggered factors in epilepsy in 11 (22.9%) patients, and acute primary CNS pathology in 5 (10.4%) patients. Sixty-two (53%) patients had a previous diagnosis of epilepsy. Etiology could not be determined in 6 (5.1%) patients. Etiologic categories in accordance with ILAE recommendations are summarized in Supplementary Table 1.

**Table 1:** Clinical and CT Perfusion Characteristics of Patients With Status Epilepticus

	Total	Normoperfusion	Hyperperfusion	Hypoperfusion	*p* value
*N* (%)	117	54 (46.2)	27 (23.1)	36 (30.8)	
Age (mean, SD)	74.6 ± 12.1	75.2 ± 12	74.2 ± 12.1	73.8 ± 12.5	0.9
Gender (F), *n* (%)	63 (53.8)	29 (53.7)	14 (51.9)	20 (55.6)	0.9
GCS ≤ 8 *n* (%)	41 (35)	20 (37)	6 (22.2)	15 (41.7)	0.2
Level of consciousness (A/So.) vs (St./C), *n* (%)	70/47 (59.8/40.2)	30/24 (55.6/44.4)	23/4 (85.2/14.8)	17/19 (47.2/52.8)	**0.006**
Focal neurologic deficit, *n* (%)	83 (70.9)	36 (66.7)	22 (81.5)	25 (69.4)	0.4
mRS preadmission ≥ 3, *n* (%)	64 (54.7)	25 (46.3)	15 (55.6)	24 (66.7)	0.1
Lactate (mean, SD) (mmol/L)	3.7 ± 3.6	3.4 ± 3.8	3.2 ± 3.2	4.6 ± 3.3	0.1
SE semiology, *n* (%)					
Motor SE	88 (75.2)	38 (70.4)	22 (81.5)	28 (77.8)	0.5
NCSE only	29 (24.8)	16 (29.6)	5 (18.5)	8 (22.2)	
Etiology, *n* (%)					
Acute symptomatic	48/111 (43.2)	25/51 (49)	12 /25 (48)	11/35 (31.4)	0.2
Structural	96 (82.1)	41 (75.9)	25 (92.6)	30 (83.3)	0.2
CVD	53 (47.7)	22 (43.1)	13 (52)	18 (51.4)	0.7
Previous epilepsy	62 (53)	30 (55.6)	13 (48.1)	19 (52.8)	0.8
CT imaging findings, *n* (%)					
Vascular lesions	46 (39.3)	20 (37)	8 (29.6)	18 (50)	0.8
Posttraumatic/postsurgical gliosis	11 (9.4)	4 (7.4)	4 (14.8)	3 (8.3)	
Periventricular leukomalacia	24 (20.5)	12 (22.2)	6 (22.2)	6 (16.7)	
Tumor	4 (3.4)	2 (3.7)	1 (3.7)	1 (2.8)	
No significant alterations	32 (27.4)	16 (29.6)	8 (29.6)	8 (22.7)	
CTA chronic vessel occlusion	9 (7.7)	3 (4.6)	0 (0)	6 (20.7)	–
CTP changes not restricted to a single vascular territory	51 (43.6)	–	22 (81)	29 (80)	0.9
Abnormal EEG findings, *n* (%)					
IED and seizure activity	44/107 (41.1)	22/50(44)	14/27 (51.9)	8/30 (26.7)	0.1
Encephalopathy and focal slowing	55/107 (51.4)	23/50 (46)	13 (48.1)	19 (63.3)	
Ictal patterns vs nonictal patterns	36 (33.6)	15 (30)	13 (48.1)	8 (26.7)	0.2
LPDs	24/107 (22.4)	9/50 (18)	11/27 (40.7)	4/30 (13.3)	**0.03**
Hemispheric agreement of pathologic EEG and CTP findings	–	–	11 (40.7)	6 (16.7)	0.7
SCC for NCSE, *n* (%)					
Definite NCSE	25 (23.4)	11 (22)	8 (29.6)	6 (20)	0.3
Possible NCSE	11 (10.3)	4 (8)	5 (18.5)	2 (6.7)	
Therapy response, *n* (%)					
Responsive SE	66 (56.4)	31 (57.4)	11 (40.7)	24 (66.7)	0.1
Refractory and super refractory SE^a^	51 (43.6)	23 (42.6)	16 (59.3)	12 (33.3)	
Use of IV anesthetics	16 (13.7)	10 (18.5)	3 (11.1)	3 (8.3)	0.4
Length of stay at hospital (*d*), mean, SD	11.5 ± 9.7	10.2 ± 8.6	17.4 ± 11.3	8.7 ± 8.2	**0.01**
Patients with SE duration ≥ 72 h, *n* (%)	27 (23.1)	10 (18.5)	12 (44.4)	5 (13.9)	**0.01**
Hospital complications, *n* (%)	62 (53)	29 (53.7)	18 (66.7)	15 (41.7)	0.1
STESS score ≥ 3, *n* (%)	75 (64.1)	36 (66.7)	15 (55.6)	24 (66.7)	0.6
In-hospital mortality, *n* (%)	26 (22.2)	8 (14.8)	9 (33.3)	9 (25)	0.2
GOS at discharge ≤ 3, *n* (%)	70 (59.8)	29 (53.7)	19 (70.4)	22 (61.1)	0.4

*A* awake, *CT* computed tomography, *CTA* CT angiography, *CTP* CT perfusion, *CVD* cerebrovascular disease, *C* coma, *d* day, *F* female, *GCS* Glasgow Coma Scale, *GOS* Glasgow Outcome Scale, *IED* interictal epileptiform discharge, *IV* intravenous, *LPD* lateralized periodic discharges, *N* number, *NCSE* nonconvulsive status epilepticus, *mRS* modified Ranken Scale, *PP* periodic pattern, *SCC* Salzburg Consensus Criteria, *SD* standard deviation, *SE* status epilepticus, *So* somnolence, *St* stupor, *STESS* Status Epilepticus Severity Score

^a^7 patients diagnosed with super-refractory SE

Statistically significant findings are marked in bold (p 0.05)

Neuroimaging (CT and MRI) revealed a presumed structural etiology for SE in over 82% of the patients (*n* = 96) (Table 1). In the CT of the brain, 46 patients (39.3%) showed signs of previously known areas of encephalomalacia due to chronic vascular lesions and 24 patients (20.5%) showed periventricular leukomalacia (Table 1). In the CTA, only 9 (7.7%) of the patients showed chronic vascular occlusive disease, and none of the patients had vascular occlusive disease due to an acute stroke.

### Timing of CT perfusion study and EEG

Most CTP studies (91/117, 82.9%) were acquired during the periictal period, within 1 h of hospital admission. EEG acquisition was delayed compared with CTP, occurring within 24 h in 39.3% (42/107), within 2 days in 38.3% (41/107), and > 2 days after admission in 22.4% (24/107).

### CT perfusion characteristics

CTP abnormalities (hyper- or hypoperfusion) were identified in 63 (53.8%) of the 117 consecutive patients included in the study. Cohen’s kappa indicated moderate-to-substantial agreement between the two raters (*κ* = 0.63, 95%CI 0.51–0.76).

Taken together, normoperfusion appears to be the most common finding in patients with SE (*n* = 54, 46.2%), followed by hypoperfusion (*n* = 36, 30.8%) and hyperperfusion (*n* = 27, 23.1%).

Patients with hyperperfusion on CTP were more frequently awake or somnolent at admission (23/27, 85.2%) compared with those with normoperfusion (30/54, 55.6%) or hypoperfusion (17/36, 47.2%) (*p* = 0.006), in whom stupor or coma occurred more often. Post hoc pairwise comparisons with Bonferroni correction (*α* = 0.017) showed significantly higher vigilance in the hyperperfusion group than in the normoperfusion group (*p* = 0.008) and the hypoperfusion group (*p* = 0.002).

Seizure duration over 72 h differed significantly between perfusion groups (Kruskal–Wallis *H* = 9.1, df = 2, *p* = 0.01). Patients with hyperperfusion on CTP (*n* = 12, 44.4%) had longer seizure durations compared with those with normoperfusion (*n* = 10, 18.5%) and hypoperfusion (*n* = 5, 13.9%) (Table 1). Post hoc pairwise comparisons with Bonferroni correction (*α* = 0.017) showed significant differences between hyperperfusion and hypoperfusion (*p* = 0.006) and between hyperperfusion and normoperfusion (*p* = 0.013).

Length of hospital stay also differed significantly between perfusion groups (Kruskal–Wallis *H* = 14.5, df = 2, *p* = 0.001). Patients with hyperperfusion (17.4 ± 11.3 days) had longer hospital stays compared with those with hypoperfusion (8.7 ± 8.2 days) and normoperfusion (10.2 ± 8.6 days). Post hoc Bonferroni tests confirmed a significant difference between hyperperfusion and hypoperfusion (*p* = 0.001).

There was no significant difference among the three groups regarding age, sex, focal neurological deficits, GCS, pre-mRS scores, serum lactate, etiology, semiology, CT lesions, therapy response, poor neurological outcome, or mortality (Table 1).

**Fig. 2:**
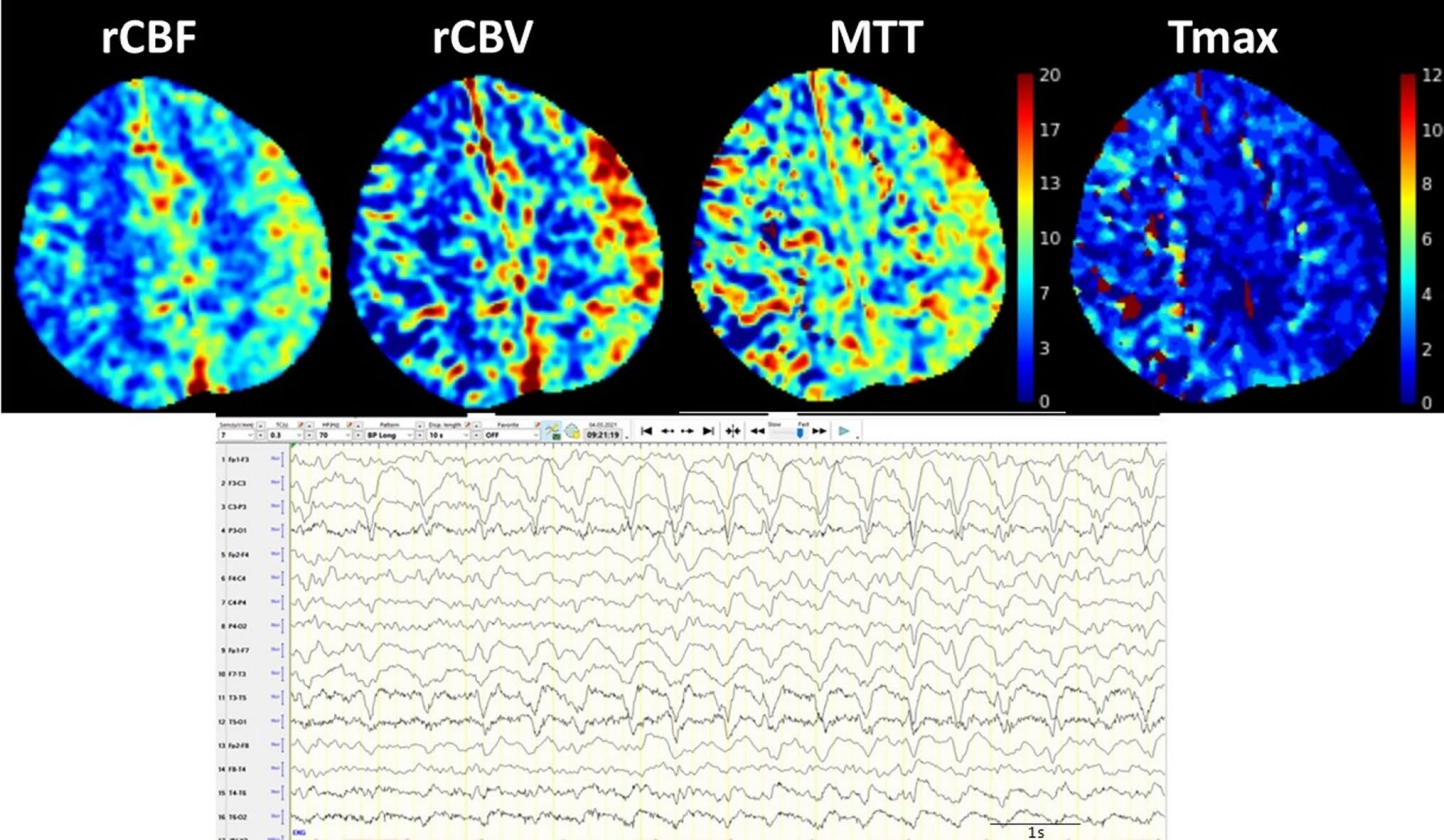
A 78-year-old patient with a history of structural epilepsy following a left middle cerebral artery (MCA) ischemic stroke presented with tonic–clonic status epilepticus, followed by aphasia and right-sided hemiparesis. Computed tomography perfusion (CTP) imaging demonstrated hyperperfusion localized to the anterior, lateral, and posterior MCA territories on the left, with increased cerebral blood volume (CBV) and cerebral blood flow (CBF). EEG performed the following day revealed recurrent seizures lateralized to the left hemisphere, consistent with nonconvulsive status epilepticus (NCSE)

Most patients have CTP changes that are not restricted to a vascular territory (*n* = 51, 80.9%) (Fig. 2). However, there is no significant difference between the hyperperfusion and hypoperfusion groups regarding vascular territory involvement (Table 1). All but one patient showed perfusion changes in the cortex, while the remaining patient had hyperperfusion in the cortex and thalamus, which were ipsilateral.

Chronic arterial occlusion (ICA or MCA) was observed in 20% of hypoperfusion patients (*n* = 6) and 4.6% of normoperfusion patients (*n* = 3), whereas none of the hyperperfusion patients were affected; differences were not statistically significant. Similarly, chronic territorial infarcts were more frequent in hypoperfusion patients (*n* = 18, 50%) than in hyperperfusion (*n* = 8, 29.6%) or normoperfusion patients (*n* = 20, 37%), without reaching significance (Table 1).

Forty of 117 patients (34.2%) underwent MRI, of whom 14 (35.0%) demonstrated periictal MRI abnormalities, including cortical or subcortical diffusion restriction and FLAIR/T2 hyperintensities. In addition, periictal MRI abnormalities were observed in 7 of 17 patients (41.2%) who underwent MRI despite having no CTP abnormalities.

### EEG characteristics

Routine EEG was performed in 107 patients, of whom 99 (84.6%) had pathological findings. In 102 patients (87.2%), ASMs were administered in the acute setting before EEG acquisition, while 32 patients (27.4%) received treatment after the detection of ictal activity in the EEG. The most frequently observed EEG pattern was focal or diffuse slowing, present in 55 patients (51.4%), whereas epileptiform activity or ictal patterns were observed in 44 patients (41.1%). We found no significant difference in the frequency of ictal (26.1% vs 38.4%) and nonictal patterns (73.8% vs 61.5%) between EEGs performed within and beyond 24 h (*p* = 0.2).

Epileptiform EEG findings (seizure patterns and epileptiform discharges) were found in 22 out of 44 patients (50%) with normoperfusion, followed by hyperperfusion (*n* = 14, 31.8%) and hypoperfusion (*n* = 8, 18.2%) (*p* = 0.1). Patients with hyperperfusion more often showed ictal patterns (*n* = 13, 48.1%) than those with normoperfusion (*n* = 15, 27.8%) or hypoperfusion (*n* = 8, 22.2%), without reaching statistical significance (*p* = 0.2). LPDs were significantly more frequent in patients with CT hyperperfusion (*n* = 11, 40.7%) than in those with normoperfusion (*n* = 9, 18%) or hypoperfusion (*n* = 4, 13.3%) (*p* = 0.03).

The classification of ictal EEG patterns according to the SCC and the corresponding CTP abnormalities is summarized in Supplementary Table 2. According to the SCC, 25 of the 36 patients with NCSE (69.4%) showed a pattern that met the criteria for “Definite SE,” while in 11 patients (30.6%), a pattern was observed that led to a diagnosis of “Possible SE.”

### Correlation between abnormal EEG and CT perfusion findings by lateralization

Out of the 63 abnormal CTP studies, only 2 patients had bilateral hypoperfusion deficits, while the rest had hemispheric perfusion abnormalities. Concordance in lateralization between CTP and EEG findings was observed in 24 patients (38%), whereas 9 patients (14.3%) had an EEG focus contralateral to the hemispheric CTP findings. The remaining patients (*n* = 20, 31.7%) showed bilateral EEG patterns; among them, 13 showed hypoperfusion and 7 showed hyperperfusion on CTP.

### Outcome

At discharge, 70 patients (59.8%) had an unfavorable outcome (GOS ≤ 3), including 26 patients (22.2%) who died. Intrahospital mortality occurred more frequently in patients with older age, potentially fatal etiology, de novo SE, hospital complications, RSE, anesthetic treatment, long SE duration (> 72 h), LPDs, seizure activity, and epileptiform discharges in EEG (Table 2) (*p* < 0.05). Acute and remote etiologies causing SE, a low GCS at onset (≤ 8), CTP abnormalities, and serum lactate levels did not play a significant role with respect to death. In the multivariate analysis, SE duration (> 72 h) (OR 9.5, 95%CI 1.9–47.4, *p* = 0.006) and RSE (OR 11.3, 95%CI 1.6–78, *p* = 0.014) were the independent predictors associated with intrahospital mortality (Table 2). A follow-up 1 year evaluation was available in 77 out of 91 cases, in whom 72 survived (96%) 1 year after SE.

**Table 2:** Variables associated with hospital mortality in patients with status epilepticus

				Univariate analysis			Multivariate analysis		
	All patients	In-hospital mortality *N* = 26	Survivals *N* = 91	Odds Ratio	95%	*p*	Odds Ratio	95%	*p*
Age^a^ (mean, SD)	74.5 ± 12.1	78.2 ± 7.9	73.5 ± 12.9			**0.001**	0.9	0.8–1	0.09
Gender (F), *n* (%)	63 (53.8)	13 (50)	50 (79.4)	0.8	0.3–1.9	0.7			
GCS ≤ 8, *n* (%)	41 (35)	9 (34.6)	32 (35.2)	0.9	0.4–2.4	1			
mRS pre- admission ≥ 3, *n* (%)	64 (54.7)	16 (61.5)	48 (52.7)	0.6	0.2–1.7	0.5			
STESS ≥ 3, *n* (%)	75 (64.1)	22 (84.6)	53 (58.2)	1.2	1.1–1.5	**0.02**			
SE semiology Motor SE, *n* (%)	88 (75.2)	20 (76.9)	68 (74.7)	1.1	0.4–3.1	0.5			
Acute symptomatic etiology *n* (%)	48 (43.2)	11 (42.3)	37 (40.1)	1.0	0.5–2.1	0.5			
CT perfusion abnormality *n* (%)	63 (53.8)	18 (69.2)	45 (71.4)	2.3	0.9–5.8	0.08			
Fatal etiology, *n* (%)	9 (7.7)	5 (19.2)	4 (4.4)	5.2	1.3–20.9	**0.01**			
Previous epilepsy, *n* (%)	62 (53)	8 (30.8)	54 (59.3)	0.3	0.1–0.7	**0.01**			
Previous SE, *n* (%)	23 (19.7)	2 (7.7)	21 (23.1)	0.3	0.06–1.3	0.09			
Hospital complication^a^, *n* (%)	52 (53)	21 (80.8)	41 (45.1)	5.1	1.8–14.8	**0.002**	0.9	0.1–4.7	0.9
RSE^a^, *n* (%)	51 (43.6)	24 (92.3)	27 (29.7)	0.008	0.15	**0.0001**	11.4	1.6–78	**0.01**
LPDs, *n* (%)^a^	24 (22.4)	11 (44)	13 (15.9)	4.1	1.5–11.2	**0.006**	2.5	0.4–13.4	0.3
PPs, *n* (%)	32 (29.9)	13 (52)	19 (23.2)	3.6	1.4–9.1	**0.01**			
Ictal patterns^a^, *n* (%)	36 (33.6)	14 (56)	22 (26.8)	3.5	1.4–8.8	**0.008**	0.3	0.04–1.8	0.2
IED and seizure activity, *n* (%)	44 (41)	15 (60)	29 (35.4)	2.7	1.1–6.9	**0.04**			
Treatment with IV anesthetics^a^, *n* (%)	16 (13.7)	9 (34.6)	7 (7.7)	6.5	2–19.4	**0.01**	1.7	0.3–7.8	0.4
SE Duration > 72 H^a^, *n* (%)	27 (23.1)	18 (69.2)	9 (9.9)	20.5	6.9–60.3	**0.0001**	9.5	1.9–47.3	**0.006**
Lactate (mmol/L) (mean, SD)	3.7 ± 3.6	3.7 ± 2.8	3.7 ± 3.7		– 1.5–1.7	0.9			

*CT* computed tomography, *GCS* Glasgow Coma Scale, *IED* interictal epileptiform discharge, *IV* intravenous, *LPD* lateralized periodic discharges, *mRS* modified Ranken Scale, *PP* periodic pattern, *RSE* refractory status epilepticus, *SD* standard deviation, *SE* status epilepticus, *STESS* Status Epilepticus Severity Score

^a^Variables involved in multivariate analysis

Statistically significant findings are marked in bold (p 0.05)

Length of hospital stay was significantly associated with acute symptomatic etiology (*p* = 0.02), absence of a history of epilepsy (*p* = 0.03), hospital complications (*p* < 0.001), RSE (*p* < 0.001), IV anesthetic therapy (*p* = 0.04), seizure activity (*p* = 0.002), and hyperperfusion on CTP (*p* = 0.01). No significant associations were observed with age, lactate levels, level of consciousness, or etiology. IV anesthetic therapy was not included in the multivariable model due to its close association with RSE. In multivariable generalized linear modeling adjusted for all other variables, only hospital complications remained independently associated with longer hospital stay [Exp(B) = 1.60, 95%CI 1.22–2.10, *p* = 0.001].

## Discussion

In our present study, we aimed to identify clinical and electrophysiological features associated with abnormal CTP in patients with SE. In our cohort, almost 54% of the patients with SE showed CTP abnormalities. Most patients exhibited normal (46.2%) or hypoperfusion (30.8%) patterns, whereas only 23.1% showed a hyperperfusion pattern. Perfusion changes as diagnosed by visual neuroradiological assessment outlining the boundaries of vascular territories were a main finding, observed in 80.9% of our patients with SE who demonstrated perfusion abnormalities. This finding is comparable to previous studies in SE [32–34]. Previous studies corroborated that the diagnostic value of hypoperfusion patterns for seizures improves when hypoperfusion extends beyond a single vascular territory [31, 35]. Our study showed no significant association between CTP patterns and different etiologies or seizure semiology.

Some earlier studies reported CTP abnormalities to be common in patients with seizures and SE, with rates as high as 77% [31, 34, 36, 37]. However, comparable normal perfusion patterns have also been described in patients with seizures, reaching frequencies of up to 65% [11, 29, 30, 33]. Variability in the results may be attributable to differences in CTP evaluation methods and the absence of standardized perfusion thresholds. In epileptic seizures, pronounced hemodynamic alterations are common; however, most of our patients had SE with prominent motor symptoms and received ASM either prehospital or upon arrival, which may explain the high number of normal perfusions. Follow-up CTP in prior studies demonstrated that hyperperfusion associated with ictal patterns often normalizes over time [37, 38]. Merli et al. demonstrated normalization of CTP in a patient with NCSE following administration of ASMs, which correlated with EEG improvement and clinical recovery [38]. In our study, it was difficult to determine whether patients were in an ictal or postictal state after CTP, primarily due to the relatively delayed performance of EEG recordings, which represent the gold standard for the diagnosis of NCSE.

It remains unclear why patients with ictal or postictal deficits in SE demonstrate either hypoperfusion or hyperperfusion. Human SPECT research indicates a rapid shift from ictal hyperperfusion to postictal hypoperfusion, though ictal hypoperfusion and prolonged postictal hyperperfusion have been observed in some cases [9, 39, 49]. Changes in cerebral perfusion may represent an adaptive response to the altered regional metabolic demands associated with periictal brain activity [33]. Epileptic activity increases neuronal metabolic demand in affected cortical regions, triggering transient hemodynamic changes through neurovascular coupling [41]. During prolonged seizures, this demand may exceed the compensatory increase in blood flow, making early hyperperfusion a likely adaptive response that protects neurons against excitotoxicity. The network inhibition hypothesis proposes that seizure propagation to the medial thalamus and upper brainstem reticular formation disrupts normal neuronal activation, causing widespread suppression in frontoparietal cortices and functional deficits such as loss of consciousness [42]. In our study, CT hypoperfusion was more frequently associated with reduced levels of consciousness, which may support this hypothesis. Additionally, a multimodal SPECT–Stereoelectroencephalography (sEEG) study demonstrated that hypoperfused regions dynamically interact with hyperperfused and normally perfused areas during seizure evolution, indicating active engagement in distributed network activity rather than passive deactivation [43].

Because hyperperfusion is rarely observed in ischemic stroke, cortical hyperperfusion on CTP can help distinguish ictal and postictal states from stroke, even in cases of arterial recanalization with cortico-subcortical perfusion patterns. In contrast, hypoperfusion is less specific, as its cortico-subcortical distribution can resemble that of ischemic stroke. A nonvascular pattern of perfusion alteration is, therefore, more suggestive of seizure than of stroke [30].

Concordance in hemispheric lateralization between CTP and EEG was observed in only 38% of patients. Seizures often involve widespread and complex brain networks, and perfusion changes may extend beyond the epileptic focus to remote brain regions, at times in the contralateral hemisphere as reported in the previous studies [34, 44]. Another possible explanation may be the relatively late performance of EEG recordings, which may have reduced the likelihood of detecting ictal activity, in addition to the rapid initiation of ASMs.

A few studies have investigated the relation between EEG patterns and the characteristics of cerebral perfusion during NCSE [45–47]. In our cohort, 45.8% of patients with LPDs exhibited CT hyperperfusion, consistent with the previous reports, supporting an association between LPDs and cortical hyperperfusion [46, 47]. No significant differences were observed in other ictal patterns, likely due to the small sample size. CTP may have relevant clinical applicability in patients within the ictal–interictal continuum [11, 38].

We found no statistical difference between definite-NCSE and possible-NCSE defined by SCC with regard to cortical perfusion. Previous studies have reported that combined thalamic and cortical hyperperfusion is a highly specific pattern of NCSE, a finding that was not observed in our study [45]. The slice thickness in CTP imaging is substantially greater than in standard structural imaging which might mask perfusion abnormalities in deep brain structures due to partial volume effects. In addition, numerous vessels course adjacent to the thalamus, particularly vessels supplying the choroid plexus, which further complicates reliable assessment of thalamic perfusion abnormalities. 58% of patients in our cohort with ictal patterns in EEG had abnormal CTP (either hypo- or hyperperfusion). Ictal EEG patterns were frequently associated with hyperperfusion on CT (36%), although this did not reach statistical significance. The proportion of hyperperfusion in NCSE varied across studies, ranging from 52.4 to 95.2% [7]. Margot et al. similarly described a lower frequency of hyperperfusion in ictal patients [30]. Small focal regions of cortical perfusion abnormalities can be missed due to limited brain coverage on standard CTP [48].

Several previous studies have reported an association between ictal patterns and CT hyperperfusion [13, 29, 30, 32, 37, 47]. However, the heterogeneity or inconsistencies in perfusion patterns and their frequencies may result from the timing of CTP relative to seizure onset and termination or from differences in seizure type and characteristics [47]. In our study, no significant association was observed between CTP patterns in patients with epileptiform disorders and those with encephalopathy as previously reported by Lee et al. [49]. However, three studies that performed EEG in the emergency setting shortly after CTP acquisition—allowing better temporal correlation between CTP and EEG findings [32, 37, 50]—reported good sensitivity and specificity for distinguishing SE from postictal conditions and other SE mimics.

According to the current guidelines, CTP can be beneficial for selecting stroke patients for reperfusion therapy in certain scenarios [51]. Although small studies suggest a potential role in diagnosing SE and NCSE, it is not currently recommended for this purpose [50]. Although CTP requires radiation exposure and contrast administration, it is widely available and rapidly performed compared to MRI in emergency settings [45]. Studies with larger cohorts encompassing diverse SE types, underlying causes, and episode durations are needed to better characterize perfusion patterns specific to each SE subtype [37].

SE classifications, underlying etiologies, and post-SE outcomes observed in our cohort were consistent with those reported in other studies [17, 52–55]. Patients with SE due to acute primary CNS pathology have been reported to have the highest reported risk of deterioration [21], but were excluded from prognostic analyses due to small numbers; thus, the null etiology–outcome association may reflect underpowering. In our study, periictal CTP changes were not associated with treatment resistance [49] or poor neurological status and mortality. Patients with hyperperfusion patterns had significantly longer SE durations and hospital stays; however, hyperperfusion was not an independent factor prolonging hospitalization. The only independent factor associated with prolonged hospitalization was the occurrence of complications. To our knowledge, the association between CTP and prognostic markers has not been systematically investigated. Another novel observation is that CT hyperperfusion may reflect disease severity and SE duration rather than irreversible brain injury, given its lack of association with reduced levels of consciousness, poor neurological outcomes, or death. In-hospital mortality was independently associated only with SE duration and treatment resistance, in line with the previous reports [17, 53, 55].

The main strength of our study is the inclusion of a cohort with detailed clinical, prognostic, and EEG data, with CTP performed predominantly within a few hours of SE onset.

This study is limited by its retrospective methodology based on a small cohort of patients. The interval between CTP and EEG recording varied among patients, preventing accurate determination of seizure chronology. Furthermore, not all patients with SE underwent CTP, and selection bias may have occurred, because only patients with suspected acute stroke were evaluated. Therefore, the observed prevalence of perfusion abnormalities may not reflect the true distribution in an unselected SE population. Follow-up CTP was not performed, and some perfusion abnormalities may reflect underlying structural lesions or vascular conditions rather than SE. In addition, CTP involves radiation exposure and IV contrast administration, which may restrict its use in some patients. Additionally, treatments given between CTP and EEG may have biased the correlation between their findings. All of our patients received routine EEG which might have missed the presence of evolving electrographic seizures or other status patterns that could have been captured on a more prolonged recording. The lack of continuous EEG (cEEG) monitoring limits accurate assessment of the duration of SE. Patients with ictal patterns may have gone undetected due to delayed EEG recordings or treatments with acute ASMs. Variability in interrater agreement may limit the general value of CTP in diagnosing NCSE in the emergency setting [11, 12, 36]. Further studies employing quantitative CTP thresholds and incorporating control groups comprising SE mimics (e.g., stroke) are needed to validate these findings.

## Conclusion

In conclusion, our study demonstrated that most of our SE patients with different etiologies had normal or hypoperfusion changes. Patients with hyperperfusion on CTP were more likely to have ictal patterns in EEG or LPDs. Functional deficits such as loss of consciousness may be more likely to be associated with CT hypoperfusion. In unclear cases with SE, CTP may aid rapid diagnostic assessment and enable earlier treatment. CTP may represent a strategy to triage patients in hospitals with limited neurophysiology services. CT hyperperfusion may be more a marker of disease severity and duration rather than of irreversible brain injury. These findings highlight the importance of multimodal integration of electrophysiological and imaging data to enhance the diagnosis and treatment of SE in emergency departments.

## Supplementary Information

Below is the link to the electronic supplementary material.

Supplementary file1 (DOCX 19 KB)

## Data Availability

Anonymized data will be shared upon request from any qualified investigator.
