# Supplementary material for: CT perfusion abnormalities in status epilepticus: associations with clinical characteristics, EEG, and outcome measures
Source: J Neurol. 2026 Jun 15;273(7):398. doi: 10.1007/s00415-026-13886-y (PMC13269492; doi:10.1007/s00415-026-13886-y)
Supplement: Supplementary file 1 — Supplementary file1 (DOCX 19 KB) [file 415_2026_13886_MOESM1_ESM.docx]

**Supplementary Table 1: Etiologic categories in accordance with ILAE recommendations [14]**

|  | N=117 | % |
| --- | --- | --- |
| **Etiology of SE** |  |  |
| Acute symptomatic | 48 | 43.2 |
| Remote symptomatic | 52 | 44.4 |
| Progressive symptomatic | 11 | 9.4 |
| **Presumed etiologic factors*** |  |  |
| Cerebrovascular Disease | 53 | 45.3 |
| Ischemic and hemorrhagic stroke | 50 | 42.7 |
| Subdural Hematoma | 3 | 2.6 |
| Small vessel cerebrovascular disease | 10 | 8.6 |
| CNS Infections | 5 | 4.3 |
| Neurodegenerative Diseases | 14 | 12 |
| Intracranial Tumors | 17 | 14.5 |
| Head Trauma | 12 | 10.3 |
| Alcohol-related | 13 | 11.1 |
| Withdrawal of or low levels of ASMs | 11 | 9.4 |
| Metabolic disturbances and systemic infections | 27 | 23.1 |
| Autoimmune disorders causing SE | 4 | 3.4 |
| Other etiologies | 3 | 2.6 |
| Unknown | 6 | 5.2 |

Other etiologies: Cerebral hypoxia or anoxia, Cortical Dysplasia, Hippocampal sclerosis. * Multiple presumed etiologies were identified in 67 patients (57.3%).

ASM: Anti-seizure medication, CNS: Central nervous system, ILAE: International League Against Epilepsy, SE: status epilepticus

**Supplementary Table 2:** **Salzburg Consus Criteria for NCSE and associated CT perfusion patterns**

|  | **Normoperfusion**  **N=15** | **Hyperperfusion**  **N=13** | **Hypoperfusion**  **N=8** |
| --- | --- | --- | --- |
| **EEG performed within 24 hours n (%)** | 6 (40) | 3 (23.1) | 2 (25) |
| **Definitive NCSE n (%)** | 11 (73.3) | 8 (61.5) | 6 (75) |
| Spatiotemporal evolution | 5 (33.3) | 5 (38.5) | 3 (37.5) |
| Subtle ictal Phenomena | 2 (13.3) | 2 (15.4) | 1 (12.5) |
| IV ASM with EEG and clinical improvement | 4 (26.7) | 1 (7.7) | 2 (25) |
| **Possible NCSE n (%)** | 4 (26.7) | 5 (38.5) | 2 (25) |
| IV ASM with EEG improvement and without clinical improvement | 2 (13.3) | 4 (30.8) | 1 (12.5) |
| Fluctuation | 2 (13.3) | 1 (7.7) | 1 (12.5) |
| **Status Patterns** **n (%)** |  |  |  |
| LPDs | 8 (53.3) | 10 (76.9) | 4 (50) |
| GPDs | 4 (26.7) | 2 (15.4) | 2 (25) |
| Rhythmic patterns | 4 (26.7) | 4 (30.8) | 4 (50) |
| Spike and Wave activity | 3 (20) | 4 (30.8) | 1 (12.5) |
| **Frequency of ictal activity n (%)** |  |  |  |
| < 1 Hz | 7 (46.7) | 7 (53.8) | 2 (25) |
| 1-2.5 Hz | 6 (40) | 5 (38.5) | 6 (75) |
| > 2.5 Hz | 2 (13.3) | 2 (15.4) | 0 (0) |
| **EEG with lateralization n (%)** | 10 (66.6) | 11 (84.6) | 7 (87.5) |
| **EEG with bilateral findings n (%)** | 5 (33.3) | 2 (15.4) | 1 (12.5) |
| **Hemispheric agreement of pathologic EEG and CTP findings, n (%)** | - | 8 (61.5) | 2 (25) |

ASM: Anti-seizure medication, CT: Computer tomography, CTP: CT Perfusion, GPD: generalised periodic discharges; IV: Intravenous, LPD: lateralized periodic discharges, NCSE: Nonconvulsive status epilepticus
